# Research on the Crack Risk of Early-Age Concrete under the Temperature Stress Test Machine

**DOI:** 10.3390/ma11101822

**Published:** 2018-09-25

**Authors:** Longlong Liu, Jianshu Ouyang, Feilong Li, Jianda Xin, Dahai Huang, Shuling Gao

**Affiliations:** 1 School of Transportation Science and Engineering, Beihang University, Beijing 100191, China; liulong4517@126.com (L.L.); auyeung@buaa.edu.cn (J.O.); lifielong@buaa.edu.cn (F.L.); 2 Department of Structures and Materials, China Institute of Water Resources and Hydropower Research, Beijing 100038, China; xinjd@iwhr.com; 3 School of Civil and Transportation Engineering, Hebei University of Technology, Tianjin 300401, China; gaoshuling@hebut.edu.cn

**Keywords:** temperature stress test machine, threshold value, complete restraint test, crack risk

## Abstract

A new temperature stress test machine (TSTM) was developed to improve temperature control accuracy and efficiency. As there is no uniform standard for the threshold value of TSTM, quite different threshold values are used in the test, which results in the influences of the threshold value on the evaluation of thermal stresses and crack risk of concrete not being determined. To illustrate the importance of an appropriate threshold value, different threshold values were used to evaluate the influences on the thermal stresses and cracking resistance of concrete specimens under a complete restraint test with semi-adiabatic temperature development. The results show that the maximum compressive stress of a concrete specimen with a threshold value 3 με was slightly larger than that of 1.5 με when the growth rate of tensile stress of the concrete specimen with the threshold value 3 με was slightly greater than that of concrete specimen with the threshold value 1.5 με. Based on the combination of crack risk coefficient and restraint degree, a new system for evaluating the crack resistance of concrete was proposed, in which different threshold values were used to estimate their influences on the crack risk of concrete. Thus, an appropriate range of threshold values could be determined.

## 1. Introduction

**Figure 1:**
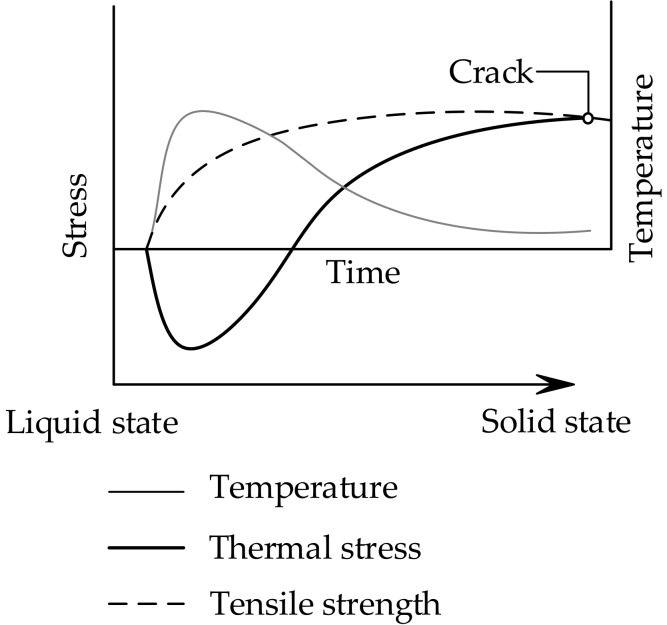
Generalized temperature and stress development in early-age concrete.

**Figure 2:**
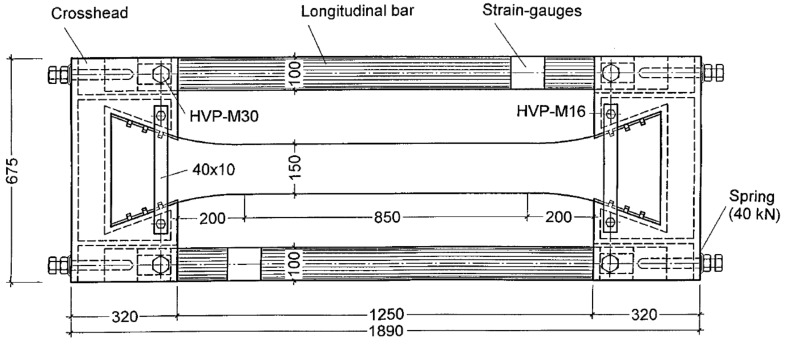
Cracking frame (mm) [9].

Cracks in young concrete not only affect the appearance and performance of the structure, but also the ability of the structure to resist external corrosive substances, which thus leads to the reduction of the durability of structures, ranging from dams, massive foundation slabs, bridge decks and piers, to beams used for buildings [1,2]. When the concrete expands during adiabatic temperature rise (temperature increases during the exothermic chemical reaction between cement and water), compressive stresses arise if the structure is restrained [3]. Then, the temperature starts to fall, and the concrete structure will contract, which gives rise to incremental tensile stresses (see Figure 1). Permanent cracks through the structure may occur when the tensile stresses exceed the tensile strength of concrete [4]. To study the cracking sensitivity of low-heat cement concrete, Springenschmid and Mangold [5,6,7] developed the cracking frame for the thermal stress and cracking sensitivity in the uniaxial restraint test, as shown in Figure 2. After a series of improvements, the cracking frame evolved into a temperature stress test machine (TSTM) when the restraint degree and temperature development could be controlled in the test. Up to now, the TSTM system has been developed with the design concepts of a closed-loop federated control, a detachable mold design, a direct measuring deformation method, and a temperature deformation compensation method [8].

The cracking frame mainly concentrated on the stress and strain development of concrete under temperature control and self-generated shrinkage deformation [10,11]. Moreover, it analyzed mechanical properties, such as the influence of elastic modulus, creep or stress relaxation, thermal expansion coefficient, thermal conductivity coefficient, and exert influences on the cracking sensitivity of concrete [9,12,13,14,15].

However, the cracking frame cannot ensure that the concrete specimen keeps its original length when the elastic modulus increases with the curing time, instead of which the incremental stiffness ratio of the concrete specimen and the steel bars reduces the restraint degree. The restraint degree $\gamma_{R}$ (%) of the concrete specimen [16] can be calculated by Equation (1).

(1) $$\gamma_{R}=\frac{100}{1+\frac{E_{C}A_{C}}{E_{S}A_{S}}}$$

where *E_C_* and *E_S_* represent the elastic modulus (MPa) of the concrete specimen and steel bar respectively, while *A_C_* and *A_S_* represent respectively the sectional area (m^2^) of the concrete specimen and steel bar on both sides of the cracking frame. As known in Equation (1), the restraint degree of the concrete specimen decreases gradually from 100% if the elastic modulus of early-age concrete increases from 0 to 50 GPa and steel bar keeps a constant value 200 GPa. The restraint degree can change from 80% to 100% if the section size of concrete specimen is the same as that of steel bar on both sides [16]. Therefore, the specimen will still be partially restrained even though the cracking frame is used for the complete restrained test.

To control the 100% restraint, threshold value [17] was applied on the deformation of concrete specimen. In the test, as the total deformation of specimen length exceeded the threshold value, the computer-controlled stepper motor [18] would push or pull the specimen to its original length [19]. Nonetheless, theoretically complete restrained specimen should be stationary and keeps its original length. As a consequence, the threshold value applied on the specimen will damage the specimen [8,17]. Measured cracking stress under this condition will reduce to some extent if the dynamic load replaces the static load [20,21]. From cracking frame to the TSTM, different threshold values have been used in the past. However, the damage degree of concrete specimen has not been specifically studied though the method has been widely used in the temperature stress test [22,23,24].

In this paper, several different threshold values were adopted to measure the stress development under the same temperature history. Compressive stresses and tensile stresses were separated to analyze the impacts of threshold values on the test results at the representative moments. A crack risk of coefficient and the restraint degree are combined to estimate the cracking risk of concrete. When the new estimation method was applied in the data from existing literature [25,26,27,28,29], it could not only evaluate the influences of the different threshold values on the cracking risk of concrete, but also determine an appropriate threshold value range.

## 2. The Temperature Stress Test

**Figure 3:**
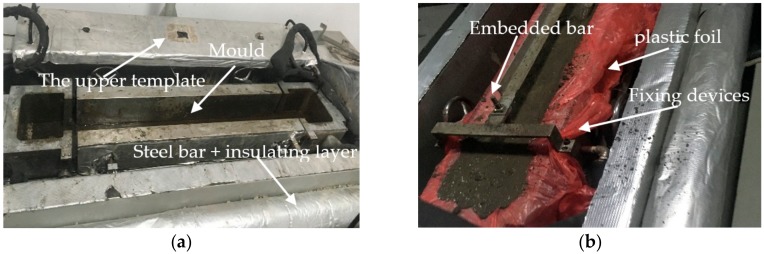
Experiment of TSTM: (**a**) the mold before pouring; (**b**) The fixing devices for embedded bar.

**Figure 4:**
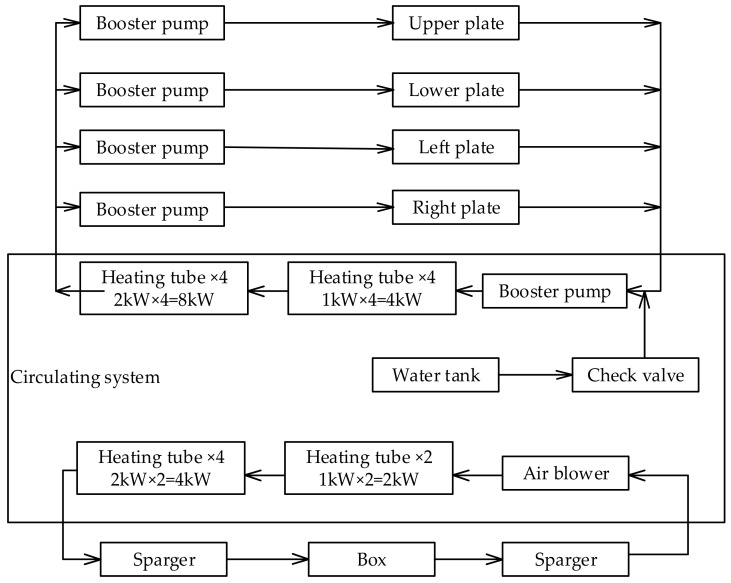
Schematic diagram of the temperature-control system.

As shown in Figure 3a,b the TSTM, developed by the China Institute of Water Resources and Hydropower Research, can work in either load-control or displacement-control mode and signals from the load sensor are digitalized with the control computer through the high precision temperature control. The temperature-control equipment combines the compressor and the heating pipe effectively. As shown in Figure 4, the temperature-control system can simulate the temperature history of mass concrete in temperature range −20 to 80 °C. The experiment result shows that the control accuracy can reach ±0.1 through the optimized design. At the time, the power consumption can reduce through the temperature-control equipment. Meanwhile, the massive concrete structures at different height have a different restraint degree [30]. Once the restraint degree is determined, a threshold value is repeated to control the concrete specimen length according to the computer-controlled system [18,31]. Based on the advanced temperature-control system and restraint degree of the concrete structures, thermal stress development and cracking temperature can be obtained to make reliable estimations of crack risk of concrete [32,33,34].

### 2.1. Test Method

The temperature stress test system was conducted to simulate the real ambient temperature and environmental conditions of structures. The isothermal conditions were also kept at a constant temperature 20 ± 1 °C with the air-conditioning. Meanwhile, the laboratory’s interior walls were faced with the thermal insulating layer to avoid the disturbance of environmental temperature. All specimens were poured and cured under the same condition, so maturity of the specimens has the same relationship with the curing age. The measured distance is 1000 mm at the middle of the concrete specimen. The total length is 1500 mm and the side length of the quadratic cross-section is 150 mm [35].

Before the concrete specimens were cast, two layers of plastic foil were snugly fitted into the mold. Apart from this sealing method, the upper template pasted the insulation layer was placed onto the specimens to obtain an enclosed condition. The temperature of the upper template was identical to that of the inner specimens, as shown in Figure 3a, which was controlled by the flowing medium (alcohol). Under this type of enclosed condition, the length deformation of concrete specimen is mainly the thermal deformation [17], and drying shrinkage can be neglected. Two embedded bars were fixed for placing displacement sensors with bolts, as seen in Figure 3b. During the pouring period, the embedded bars were suspended in the mold. After the setting time (about 10 h) of concrete, the devices for fixing embedded bars would be removed.

**Figure 5:**
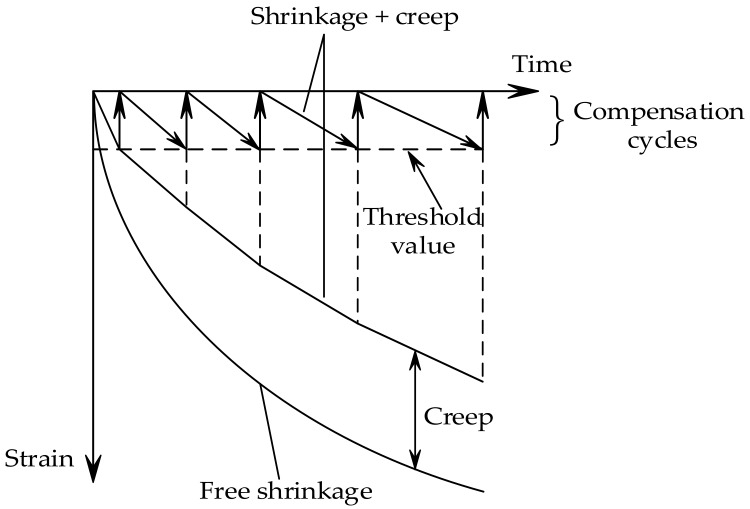
The restrained shrinkage test.

One end of the restrained specimen was connected to a stepping motor through a universal joint, and the other end was fixed. The specimen would drive the universal joint to move when the specimen length changed with the temperature history. Then the stepping motor would push or pull the universal joint to its original position when the absolute value of the total strain of the restrained specimen exceeded the threshold value [17,25,36], as shown in Figure 5. A compensation cycle is repeated at increments of threshold value to recover the shrinkage strain [18,31,37]. Two threshold values 1.5 and 3 μm (1.5 and 3 με for a 1000-mm long specimen adopted in this study) were adopted to investigate the thermal stress development and crack resistance of concrete.

### 2.2. Concrete Mixture Proportions

The temperature stress tests were performed for the low-heat cement concrete, which was used in the dam structures, including low-heat Portland cement P·LH 42.5, the first-grade fly ash (35% replacement cement by fly ash), nature sand and crushed limestone (maximum grain size of 20 mm). Table 1 shows the concrete mixture proportions, in which the concrete mixture had a paste volume fraction of 0.5 and a w/c ratio of 0.77. The mechanical properties of concrete were tested according to the Chinese code [38], in which the compressive strength of concrete at 28 days is 24.9 MPa and the direct tensile strength is 2.86 MPa at 28 days.

### 2.3. Temperature History and Free Deformation

**Figure 6:**
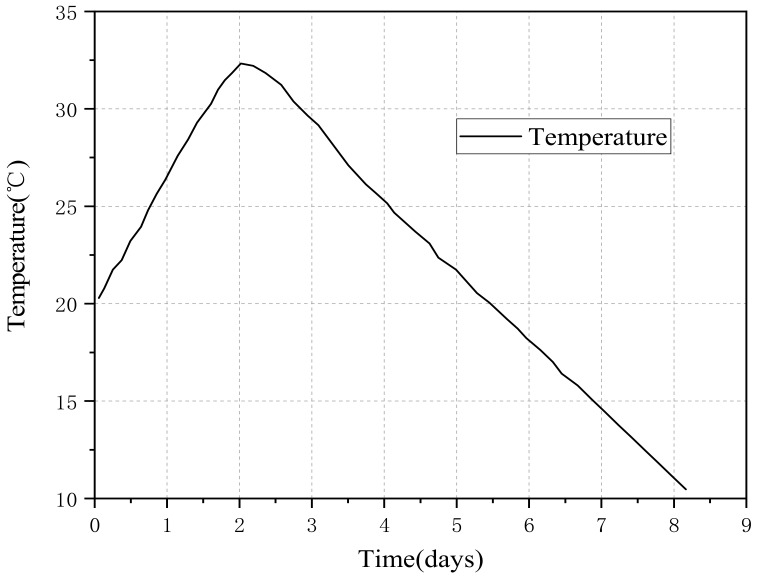
Temperature development under semi-adiabatic condition.

**Figure 7:**
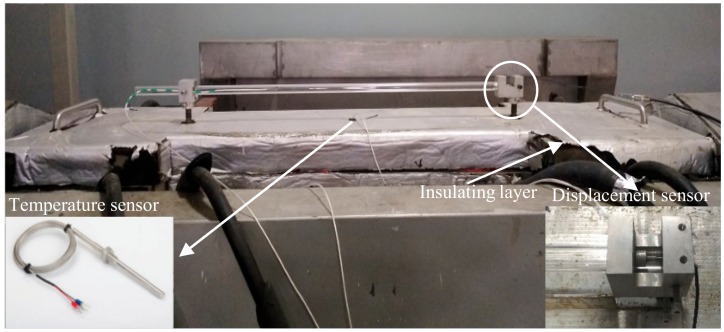
Measured devices of the TSTM.

Figure 6 shows the temperature development. The concrete specimen temperature increased from 20 °C to above 32 °C at 0.25 °C/h within 48 h, and then cooled down until the specimen lost its bearing capacity. As shown in Figure 7, the temperature was measured inside of the specimens. The copper tube was inserted into the concrete after the concrete specimen was poured into the mold. Then the temperature sensor can be put inside the copper tube.

In addition to two layers of plastic foil snugly fitted into the mold when the concrete specimens were cast, the cover was made of temperature-control template plus insulating layer, as shown in Figure 7. The previous experiment results showed that this type of enclosed method is sufficient to suppress the deformation of drying shrinkage [39]. The free deformation was directly measured by the displacement sensor through the distance between two embedded bars shown in Figure 7. The measured free deformation was the sum of thermal deformation and autogenous volume deformation. The autogenous deformation was so small that it can be neglected. Therefore, the free deformation can also be calculated by the temperature expansion coefficient (7.5 × 10^−6^ m/°C) and temperature difference.

### 2.4. Restrained Deformation and Stress Evolution of Concrete Specimen

**Figure 8:**
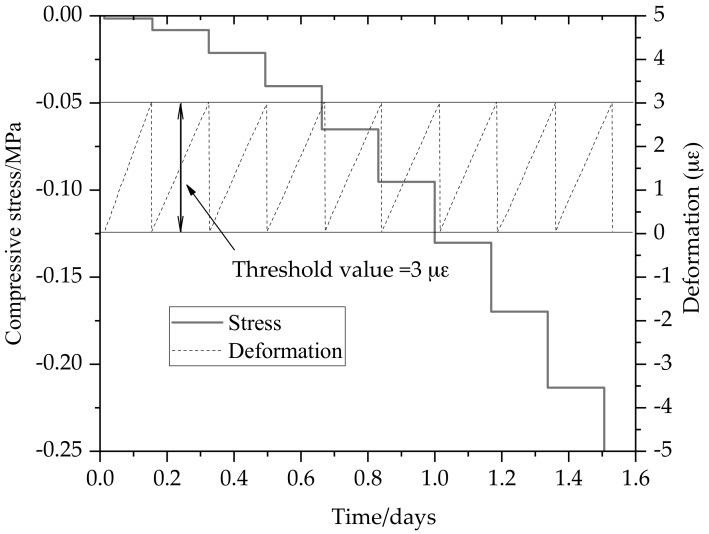
Compensation deformation cycles and stress evolution for restrained concrete specimen.

As shown in Figure 8, the compressive stresses started to increase when the specimen was restrained due to thermal dilation. At the initial loading stage, the stress increased by a threshold value is less than 0.025 MPa. In general, the compressive stress in its first few hours can be neglected because the elastic modulus of concrete is very small. Therefore, the stress measured by the load sensor should reach 0.01 MPa at least. The displacement sensor was reset [26]. Since the elastic modulus of the specimen is very small at early age and the stiffness of steel bars on both sides is far greater than that of early-age concrete, the restraint degree can reach 100% when the gripped end remains static. The compressive stress increased 0.05 MPa for a threshold value after one day, and the compressive stress increased per step steadily. With the growth of temperature, the deformation of the concrete specimen is mainly the thermal deformation. The step motor pushed the concrete specimen to its original length with the enhancement of compressive stresses before the temperature reached the maximum. In the experiment, the applied load, the deformation of the restrained and free concrete specimen, the temperature development and the step motor displacement were measured.

## 3. Results and Discussions

### 3.1. Thermal Stress Development under Different Threshold Values

**Figure 9:**
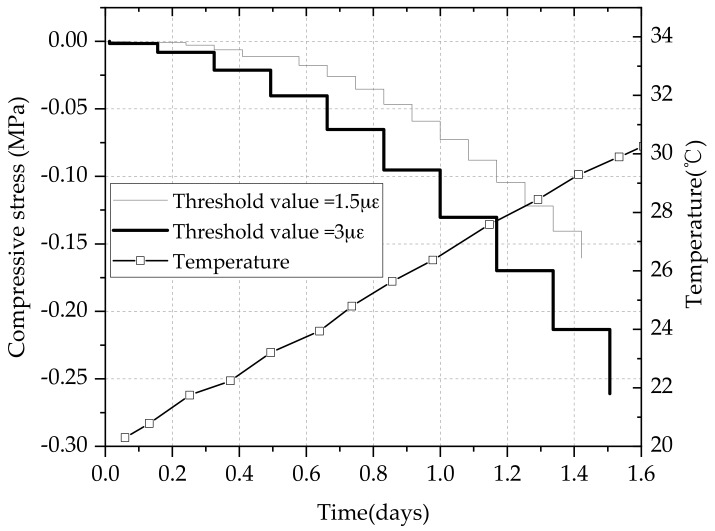
Compressive stress under different threshold values.

As shown in Figure 9, different threshold values have influences on the compressive stress when the heating rate is 0.25 °C/h. The same temperature rise should make identical stress history, but the compressive stress under different threshold values show a little difference as the elastic modulus of concrete specimen increases quickly at early age. 24 h later, the compressive stress of 3 με increases faster than that of threshold value 1.5 με. The former compressive stress has reached −0.125 MPa while the later −0.075 MPa at this point. The compressive stress difference is about 0.1 MPa between the threshold value 3 and 1.5 με at the age of 36 h. This phenomenon is caused by the rapid growth of elastic modulus of concrete at early age. The stress difference between different threshold gradually reduces until the growth rate of the elastic modulus falls.

**Figure 10:**
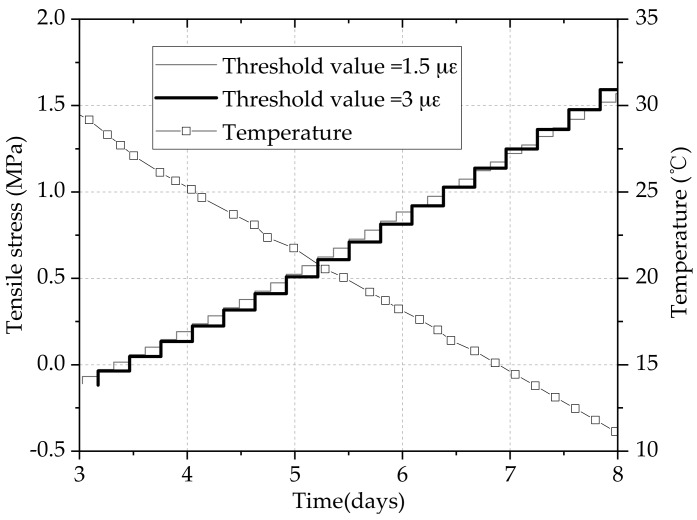
Tensile stress under different threshold values.

To compare the effects of different threshold values on thermal stresses, the heating and cooling stages are separated to analyze. The restraining tensile stress development during the cooling stage is presented in Figure 10. Compared with the compressive stage, this early contraction during the cooling stage shows up as greater tension, which is more uniform than the compressive development. When the forced state of concrete alters from compression to tension, the tensile stress under the threshold value 3 με was significantly lower than that of specimen under the threshold value 1.5 με. This is because the tensile stress that a specimen under the threshold value 3 με starts to generate based on greater compressive stress. Then the tensile stress of concrete specimen under the threshold value 3 με began to exceed the concrete specimen under the threshold value 1.5 με at the age of 6 days. Therefore, the growth rate of stress under a higher threshold value will be greater no matter it is in compression or tension phases. Nevertheless, the stress difference at high stress levels under different threshold values showed a little difference when the elastic modulus of concrete kept a relatively stable value.

### 3.2. Evaluation of Crack Risk Coefficient

For the early-age concrete specimen, the frequent compression and tension will damage the specimen [8], but the damage degree has not been determined at present. Only when the concrete specimens show the non-linear stress-strain relationship, the relative stress levels (*σ*/*f_ct_*) exceed 0.5 [40]. This is mainly because of the increase of the micro-cracks [41]. However, these explanations are just for the static test. If the dynamic load replaces the static load, these growth micro-cracks may lead to earlier failure with incremental threshold value [42,43,44].

To illustrate the influences of different threshold values on the tensile capacity of the concrete specimen [17], the relative stress level (*σ_crack_*/*f_t_*) is taken as the evaluation criterion that represents the ratio between the cracking stress *σ_crack_* and the tensile strength of concrete specimen. The following Equation (2) was defined as the crack risk coefficient *η*_1_ of concrete [45].

(2) $$\eta_{1}=\frac{\sigma (t)}{f_{t}(t)}$$

The complete restraint stress *σ_fix_(t)* can be measured by the TSTM while the restraint degree *γ_R_* [4] from different position of massive concrete structures can be combined with the crack risk coefficient to obtain a new evaluation method *η*_2_.

(3) $$\gamma_{R}=\frac{\sigma (t)}{\sigma_{fix}(t)}$$

(4) $$\eta_{2}=\frac{\sigma_{fix}(t)}{f_{t}(t)}\cdot \gamma_{R}$$

where *σ(t)* and *f_t_(t)* represent the stress and tensile strength at time *t* respectively, while *σ_fix_(t)* denotes the 100% restraint stress at time *t*.

Now the specimen is 100% restraint, so

(5) $$\eta_{2}=\frac{\sigma_{fix}(t)}{f_{t}(t)}$$

*η*_2_ can be used to evaluate the influences of different threshold value on the crack risk of concrete in this study. Apart from that, it can also be used to estimate the risk of cracking.

**Figure 11:**
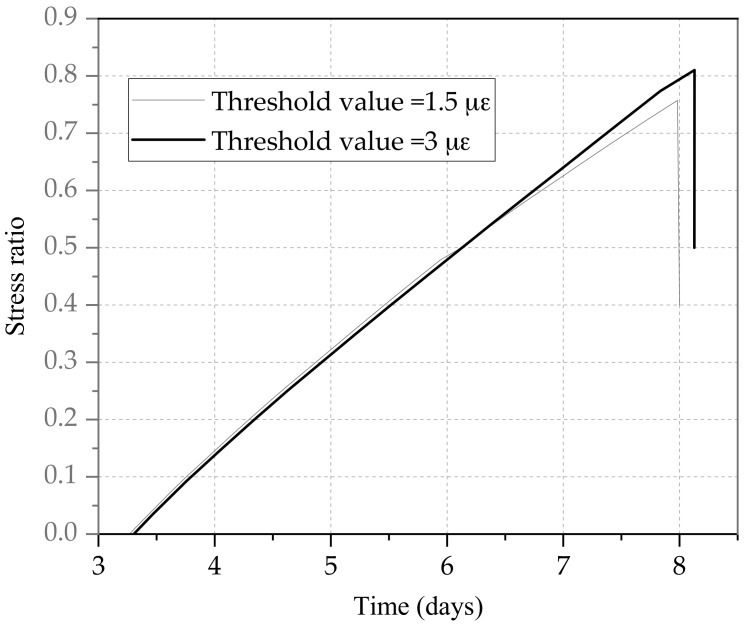
The ratios of stress under different threshold values.

As shown in Figure 11, the concrete specimen with a threshold value of 1.5 με was destroyed at a stress ratio of 0.75, while the other with a threshold value of 3 με was destroyed at a stress ratio of 0.8. In terms of cracking stress, the damage degree of specimen with a threshold value 1.5 με was slightly greater than that of specimen with a threshold value 3 με, because the damage degree decreased with the greater cracking stress. The stress history of the specimen with a threshold value 3 με was more stable as a whole.

**Figure 12:**
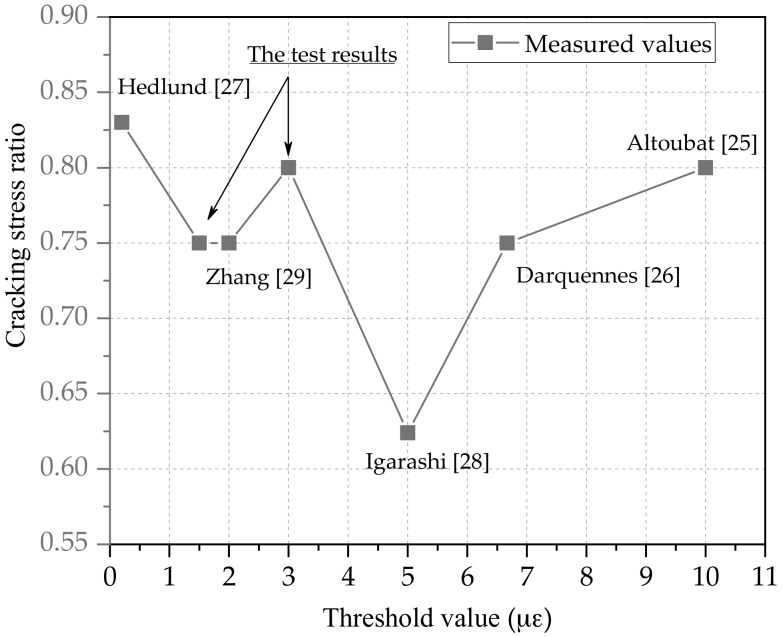
Cracking stress ratio under different threshold value.

Altoubat [25] studied the 100% restraint concrete specimen with a threshold value 10 με. The cracking stress ratio of 7 set of specimens were among 0.76–0.91 and the average value was 0.8. Using 8 groups of specimens with different mix ratios, Zhang [29,46] did the same research with a threshold value 2 με, The cracking stress ratios were among 0.67–0.82 and the mean value was 0.75. Concerning on the cracking stress ratio with 100% restraint degree, Hedlund [27] adopted a smaller threshold value 0.2 με.The experiment results were among 0.72–0.94 and the average value was 0.83. Igarashi [28] found that cracks occurred with a threshold value 5 με when the ratio between the restraining stress and the tensile strength approached 50%. Darquennes [26] used a threshold value 6.67 με with 100% restraint degree, and the average cracking stress ratio was 0.75. Key information about the tests are given in Table 2 and Table 3, and the average value (${\bar{\eta }}_{2}$) of cracking stress ratio are plotted in Figure 12. When the threshold value changed from 1.5 to 3 με, the cracking stress ratio of specimens were between 0.75 and 0.8. By contrast, when the threshold value changed from 0–1.5 or 3–10 με, the cracking stress ratio of specimens had a larger discrete behavior.

The cracking capacity of early-age concrete is also affected by the factors such as temperature development, water-cement ratio, and structure size, which will affect the mechanical properties of early-age concrete, and a series of studies have been conducted [11,29,32]. These factors influencing the new estimation method can be studied in the future. This paper mainly investigates the influence of threshold values with a TSTM method on cracking evaluation of early-age concrete. Therefore, to eliminate the influence of other factors on the test results, two threshold values (1.5 and 3 με) were adopted to investigate the crack resistant of early-age concrete under the same condition.

### 3.3. Effect of Threshold Value on the Whole Stress History

**Figure 13:**
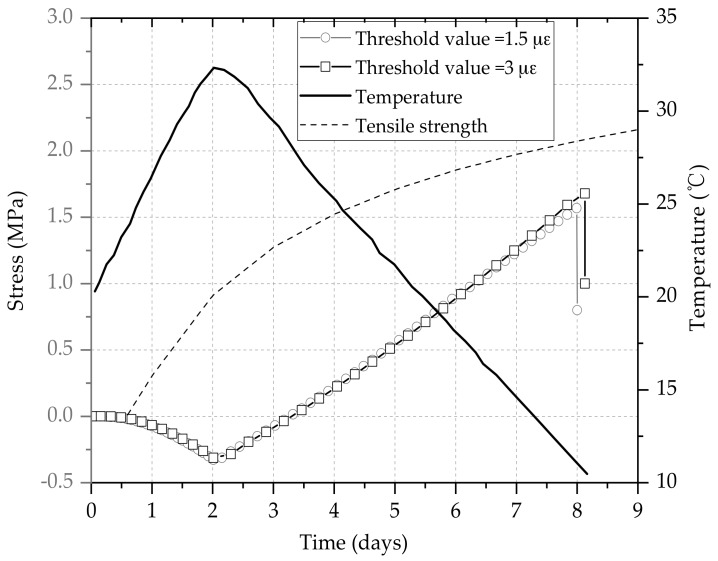
Restrained stress development under semi-adiabatic condition.

As shown in Figure 13, the adiabatic temperature-rise and -cooling history is simulated and the stress development was basically the same as the different threshold values. The tensile strength of concrete specimen increased rapidly from 3 days to 6 days, then the growth rate was slowing down.

As observed in the figure, the initial tensile stress rises at a growth rate of about 0.015 MPa/h when the cooling rate is 0.15 °C/h. The stress and deformation of specimens showed a linear stress-strain relationship and the stress and temperature could also be in a simple linear relationship, i.e., 0.1 MPa/°C. When the cracks occurred, the tensile stress had not reached the tensile strength.

As outlined above, different threshold values have different effects on the compressive and tensile stresses during the heating and cooling phases. Figure 13 shows the influences of different threshold values on the whole stress development. It is found that different threshold values have similar influences on the whole stress development. This phenomenon is mainly because that the elastic modulus and tensile strength of concrete increased exponentially with curing age. The stress was applied at different time when the elastic modulus increased, so the stress could result in differences in each threshold value. However, when the two adjacent threshold values were applied at very close time, the elastic modulus had a slight increase. Therefore, the stress increment with different threshold values during the same time step was about equivalent.

## 4. Conclusions

The temperature-control system in TSTM has been improved, which can raise and lower temperature with very high precision. The setup could quickly control the temperature of concrete specimens with a high-accuracy temperature measurement. Different threshold values have different influences on the thermal stress. No matter if it is in the temperature-rise or temperature-fall period, the compressive/tensile stress growth rate of threshold value 3 με was slightly higher than that of threshold value 1.5 με. This is mainly because of the rapid growth of elastic modulus of concrete at early age. Under semi-adiabatic temperature history, the maximum compressive stress of concrete specimens with threshold values of 1.5 and 3 was −0.33 MPa and −0.31MPa, while the cracking stress ratio was 1.61 MPa and 1.71 MPa, respectively. Different threshold values have mild influences on the whole thermal stress development when the elastic modulus starts to have a basically stable value.

Compared with the experiment results of TSTM, the cracking stress ratio of concrete specimens with threshold value 1.5–3 με was centralized between 0.75–0.8 while the cracking stress ratio of concrete specimens with threshold value 0–1.5 and 3–10 με was relatively discrete. The new evaluation method $\eta_{2}$ could not only be used to estimate the risk of cracking, but also represented the residual bearing capacity of the specimen. As a simplified method, $\eta_{2}$ should combine with different restrained degree to evaluate the crack risk of concrete in the future.

## Figures and Tables

**Table 1:** Concrete mixture proportions.

Cement	Water	FA	Coarse	Fine	Superplasticizer	Air Entraining Agent
216	166	116	844	1118	1.992	0.053

**Table 2:** Cracking stress ratio under different threshold values. * denotes aborted data.

Source	Threshold (με)	W/C	W/B	Age (h)	Stress (MPa)	Direct Tensile Strength (MPa)	Stress/Strength (*η*_2_)
Hedlund [27]	0.2	0.3	0.27	128.3	3.8	4.58	0.83
	0.2	0.3	0.27	83.6	3.41	4.1	0.83
	0.2	0.3	0.27	75.8	2.36	3.1	0.76
	0.2	0.34	0.31	110.5	3.12	3.95	0.79
	0.2	0.34	0.31	118.5	3.58	4.42	0.81
	0.2	0.34	0.31	139.4	4.03	4.58	0.88
	0.2	0.42	0.4	97.74	2.32	2.68	0.865
	0.2	0.42	0.4	192.6	2.79	3.42	0.816
	0.2	0.42	0.4	215.8	2.17	2.66	0.816
	0.2	0.42	0.4	72.2	2.39	3.32	0.72
	0.2	0.42	0.4	102.4	3.05	3.63	0.84
	0.2	0.42	0.4	122.9	2.10	2.53	0.83
	0.2	0.42	0.42	94.42	2.57	2.74	0.94
	0.2	0.43	0.43	136.2	2.38	2.87	0.83
Zhang [29]	2	0.41	0.35	-	2.76	3.42	0.81
	2	0.5	0.35	-	2.38	2.91	0.82
	2	0.35	0.35	-	2.28	3.04	0.75
	2	0.3	0.3	-	3.26	4.26	0.76
	2	0.29	0.29	-	2.54	3.77	0.67
	2	0.32	0.29	-	2.5	3.45	0.72
	2	0.3	0.27	-	3.32	4.29	0.77
	2	0.38	0.35	-	2.68	3.84	0.7
Igarashi [28]	5	0.25	0.25	100.4	2.98	4.44	0.672
	5	0.28	0.25	100.7	3.1	5.74	0.54
	5	0.33	0.33	144.0	2.87	4.35	0.66
	5	0.37	0.33	142.0	1.4	4.83	0.29*
Darquennes [26]	6.7	0.44	0.44	79.2	3	4.0	0.75
	6.7	0.44	0.44	110.4	3	4.0	0.75
Altoubat [25]	10	0.32	0.4	69.5	1.76	2.325	0.757
	10	0.4	0.35	144.7	2.13	2.649	0.804
	10	0.5	0.35	159.5	1.78	2.214	0.805

**Table 3:** Details of restrained specimens under different threshold values.

Design Parameters	Hedlund [27]	Zhang [29]	Igarashi [28]	Darquennes [26]	Altoubat [25]
Threshold value (με)	0.2	2	5	6.67	10
Section size (mm × mm)	150 × 150	100 × 100	40 × 40	100 × 100	76.2 × 76.2
Specimen length (mm)	1000	1000	1000	1000	1000
Measured distance (mm)	1000	1000	1000	750	1000
Average of stress ratio (${\bar{\eta }}_{2}$)	0.83	0.75	0.624	0.75	0.8
